# Characterization of a Novel Polyomavirus Isolated from a Fibroma on the Trunk of an African Elephant (*Loxodonta africana*)

**DOI:** 10.1371/journal.pone.0077884

**Published:** 2013-10-18

**Authors:** Hans Stevens, Mads Frost Bertelsen, Steven Sijmons, Marc Van Ranst, Piet Maes

**Affiliations:** 1 Laboratory of Clinical Virology, Department of Microbiology and Immunology, Rega Institute, KU Leuven, Leuven, Belgium; 2 Center for Zoo and Wild Animal Health, Copenhagen Zoo, Frederiksberg, Denmark; University of Kansas Medical Center, United States of America

## Abstract

Viruses of the family *Polyomaviridae* infect a wide variety of avian and mammalian hosts with a broad spectrum of outcomes including asymptomatic infection, acute systemic disease, and tumor induction. In this study a novel polyomavirus, the African elephant polyomavirus 1 (AelPyV-1) found in a protruding hyperplastic fibrous lesion on the trunk of an African elephant (*Loxodonta africana*) was characterized. The AelPyV-1 genome is 5722 bp in size and is one of the largest polyomaviruses characterized to date. Analysis of the AelPyV-1 genome reveals five putative open-reading frames coding for the classic small and large T antigens in the early region, and the VP1, VP2 and VP3 capsid proteins in the late region. In the area preceding the VP2 start codon three putative open-reading frames, possibly coding for an agnoprotein, could be localized. A regulatory, non-coding region separates the 2 coding regions. Unique for polyomaviruses is the presence of a second 854 bp long non-coding region between the end of the early region and the end of the late region. Based on maximum likelihood phylogenetic analyses of the large T antigen of the AelPyV-1 and 61 other polyomavirus sequences, AelPyV-1 clusters within a heterogeneous group of polyomaviruses that have been isolated from bats, new world primates and rodents.

## Introduction

Members of the family *Polyomaviridae* are small viruses characterized by a non-enveloped icosahedral capsid and a circular double-stranded DNA genome of approximately 5000 base pairs (bp). Early and late genes are transcribed bi-directionally, starting from a short non-coding regulatory region. Early genes encode two or three proteins, designated tumor antigens, which participate in viral genome replication and transformation of infected cells. The late genes encode the major and minor capsid proteins VP1, VP2, and VP3 [1]. In addition, some of the primate and human polyomaviruses encode an additional non-structural multifunctional protein, the so-called agnoprotein [2]. Most mammalian polyomaviruses cause subclinical infections with life-long persistence in their natural immune competent hosts. However, when the host immunity is compromised, the virus can reactivate and cause disease. The International Committee on Taxonomy of Viruses officially lists thirteen species as polyomaviruses, but a new proposal increases that number to 28 and divides the family into three genera: two containing mammalian viruses (genus Orthopolyomavirus, and genus Wukipolyomavirus) and one containing avian viruses (genus Avipolyomavirus) [3]. In humans, so far 13 different polyomaviruses have been described. The best-studied human polyomaviruses BK virus and JC virus are associated with severe disease in immunosuppressed patients [4,5]. WU and KI virus have been found in patients with respiratory tract infections [6,7], and Merkel cell polyomavirus was found in patients with Merkel cell carcinoma, a rare aggressive skin cancer [8]. The *Trichodysplasia spinulosa*-associated polyomavirus was discovered in proliferative skin lesions, termed *trichodysplasia spinulosa*, sometimes seen in immunosuppressed patients [9]. Two other polyomaviruses, HPyV-6 and HPyV-7, commonly inhabit healthy human skin are as yet not associated with human disease [10]. HPyV-9 was cultured from the blood of immunosuppressed patients [11]. More recently, several polyomaviruses (MX, MW, STL and HPyV-12) have been isolated out of human feces or biopsies of gastrointestinal tract organs [12-15].

Polyomaviruses are widely distributed among mammalian and avian species and besides humans they can infect monkeys, cattle, rabbits, rodents, bats, and a broad variety of bird species, mainly in a host-specific manner. Initially, most of the polyomaviruses were discovered by screening tissue cultures for viral contaminants [16] and more recent studies describe the detection of new polyomaviruses in various wild or captured animal species [17-25]. These studies indicate that there could still be many unknown polyomaviruses in wild animals.

In this article we describe the isolation and characterization of a novel polyomavirus found in a protruding hyperplastic fibrous lesion on the trunk of an African Elephant (*Loxodonta africana*), the African elephant polyomavirus 1 (AelPyV-1).

## Materials and Methods

### Sample Origin and Pathology

**Figure 1:**
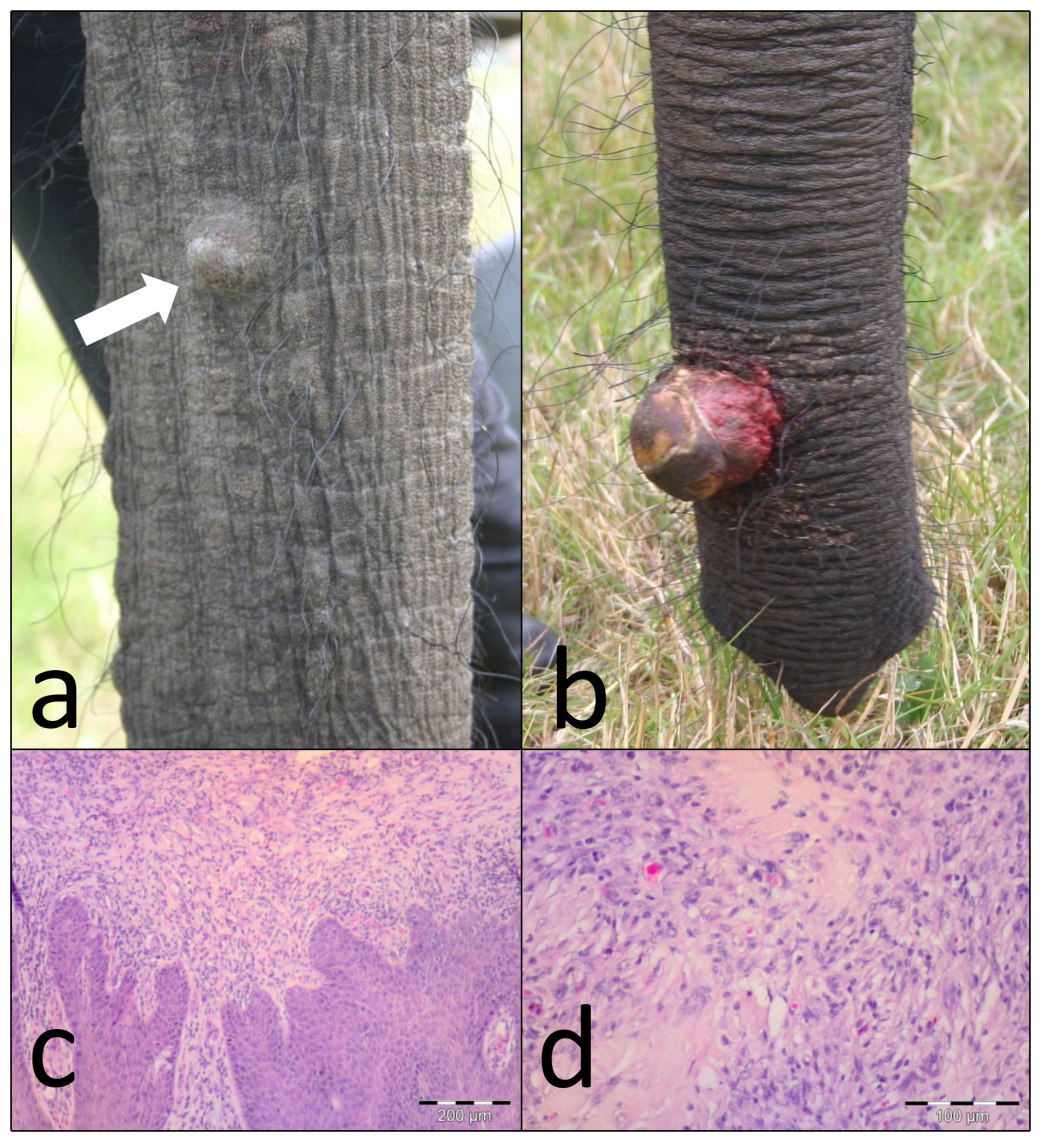
Lesions found on the trunks of the elephants. (a) Nodular lesion as seen on the trunks of three elephants; (b) Protruding ulcerated fibroma, from which the biopsy was taken; (c) Interface between the fibroblastic mass and proliferative dermis; (d) Whirling fibroblasts with prominent collagen deposits.

Three African elephants from a European circus developed multiple processes on their trunks over a period of months. Lesions started as plaques or nodules of a few mm and grew in size up to 7 cm. Some were flattened, others pedunculated (Figure 1A and 1B). In one female, two entire masses were surgically removed. These masses were 3 and 7 cm in diameter respectively, and had a narrow (2 cm) base. Histologically, the masses were characterized by dermal hyperplasia overlying massive proliferation of fibroblasts (Figure 1C and 1D). These fibroblasts were arranged in whorls or disorderly bundles intermixed with collagen and entrapped muscle cells. Superficially, there was hemorrhage and focal heterophilic inflammatory infiltration. Biopsies were frozen at -80° C immediately after surgical removal. Whole genomic DNA was extracted from the frozen biopsy with the QIAamp DNA Blood Mini kit (Qiagen, Hilden, Germany), according to the manufacturer’s tissue protocol.

This work did not involve any animal experiments. The tissue lesions were surgically removed under strict anesthesia by licensed veterinarians of the Copenhagen Zoo in the best interest of the animals and were removed to avoid further complications. Every effort was made to minimize suffering. These lesions were not removed for the purpose of research.

### Rolling Circle Amplification

For the amplification of the complete genomic DNA, multiply primed RCA was performed on extracted DNA by using the TempliPhi 100 Amplification kit (GE Healthcare Life Sciences, Pittsburg, PA, USA). A protocol was used which is optimized for the amplification of circular DNA viruses [26,27]. Briefly, the extracted DNA was combined with TempliPhi sample buffer containing exonuclease-protected random hexamers. Subsequently, the samples were denatured and a mixture of TempliPhi reaction buffer, TempliPhi enzyme containing the phi29 DNA polymerase, and extra dNTPs were added to the samples. The reactions were incubated at 30° C and left overnight after which the reaction was stopped by inactivation of the enzyme at 65° C. To investigate whether polyomavirus DNA was amplified, the RCA products were digested with BamHI, EcoRI, HindIII, SalI, and XbaI and put on an agarose gel to check for the presence of a DNA band consistent with full length polyomavirus DNA (~5.5 kb) or multiple bands with sizes adding up to this length. The remaining RCA product was precipitated to remove excess phi29 DNA polymerase, buffer, dNTPs and random hexamers. The final DNA concentration and quality were determined using an Eppendorf BioPhotometer.

### 454: Next-Generation Sequencing of the African Elephant Polyomavirus genome

The complete genomic sequence of the AelPyV-1 was determined using the 454 GS FLX platform. Total DNA was fragmented to an average length of 400 bp using a Covaris E210 system (Covaris®). DNA fragments were end-repaired, 3’-adenylated, ligated to adapters (GS FLX Titanium Rapid Library MID Adaptors kit, Roche Applied Science, Penzberg, Germany) and size-selected (>350 bp) using the SPRIworks Fragment Library System II (Beckman Coulter Genomics, Essex, UK). The quality of the library was evaluated by using a high-sensitivity DNA chip on a model 2100 Bioanalyzer (Agilent Technologies, New Castle, DE, USA). Libraries were quantified with the TBS-380 Mini-Fluorometer (Promega, Madison, WI, USA) in order to pool them at equimolar concentrations. Prior to sequencing, clonal amplification was performed during an emulsion based PCR (GS FLX Titanium emPCR Kit, Roche Applied Science). Sequencing was performed using the GS FLX Titanium Sequencing Kit (Roche Applied Science). Following sequencing, processing of the raw sequence data was performed with the CLC Genomics Workbench v6.0.5 software (CLC bio, Aarhus, Denmark).

Sequencing of the library created from the whole-genome amplified sample resulted in 359 successful reads, representing 145,230 bases. Reads were assembled into one contig 5729 bp in size. The sequence data obtained by 454 sequencing were used to design primers for classic Sanger sequencing. The Sanger sequencing method was used to verify the sequence data acquired by 454 sequencing. Sanger sequencing was performed on an ABI Prism 3120 Genetic Analyzer (Applied Biosystems, Life Technologies, Carlsbad, CA, USA). The chromatogram sequencing files and contigs were prepared and compared with the 454 sequencing data by using the CLC Genomics Workbench v5.5.1 software.

### DNA and Protein Sequence Analysis

The CLC Genomics Workbench software was used for the open reading frame (ORF) analysis. Multiple nucleotide sequence alignments were constructed using MAFFT v7.012. Maximum likelihood trees were calculated by the heuristic search method using random addition, tree-bisection reconnection for swaps, and branch swapping by the Rogers-Swofford approximation algorithm with PAUP* v4.0b10. jModeltest 2 was used to identify the evolutionary model that best fits the data; the phylogenetic model GTR+G+I was selected to calculate the likelihood parameters, with a gamma distribution shape of 0.922. Bootstrap analysis based on 10000 replicates was used to estimate the statistical support for the branching pattern.

Amino acid phylogenetic trees were generated in Paup* by the neighbor joining method using the JTT substitution matrix. Bootstrap analysis based on 10000 replicates was used to estimate the statistical support for the branching pattern.

### Nucleotide sequence accession number

The nucleotide sequence of AelPyV-1 has been submitted to NCBI GenBank under accession number KF147833.

## Results and Discussion

We used a rolling circle amplification method (RCA) followed by a 454-pyrosequencing strategy to identify a novel polyomavirus in a biopsy from a fibroma on the trunk of an African elephant, the African elephant polyomavirus (AelPyV-1). The multiply primed RCA technique was used to amplify the complete AelPyV-1 genome. This technique has proven to be very useful for the detection of novel circular DNA viruses. Not only novel polyomaviruses but also novel papillomaviruses and even a possible novel virus family, the bandicoot papillomatosis carcinomatosis viruses, were discovered and amplified using this technique [28-31]. The RCA technique makes use of bacteriophage phi29 DNA polymerase, the strand displacement capacity of this polymerase produces a high molecular-weight DNA, containing repeated copies of the whole AelPyV-1 genome. Moreover, the enzyme has a proofreading activity to guarantee a low error rate during the amplification [28]. After the amplification of the extracted whole-genomic DNA, the resulting products were subjected to a restriction enzyme analysis. Cutting the RCA product with XbaI and HindIII resulted in 1 band of approximately 6 kb, cutting with BamHI produced 2 bands of approximately 5.3 kb and 7 kb, and finally EcoRI produced 3 bands of approximately 2.3 kb, 2 kb and 1.7 kb in size. Although rather large, the length of the produced fragment, or sum of lengths, was indicative for the presence of a putative novel polyomavirus.

**Figure 2:**
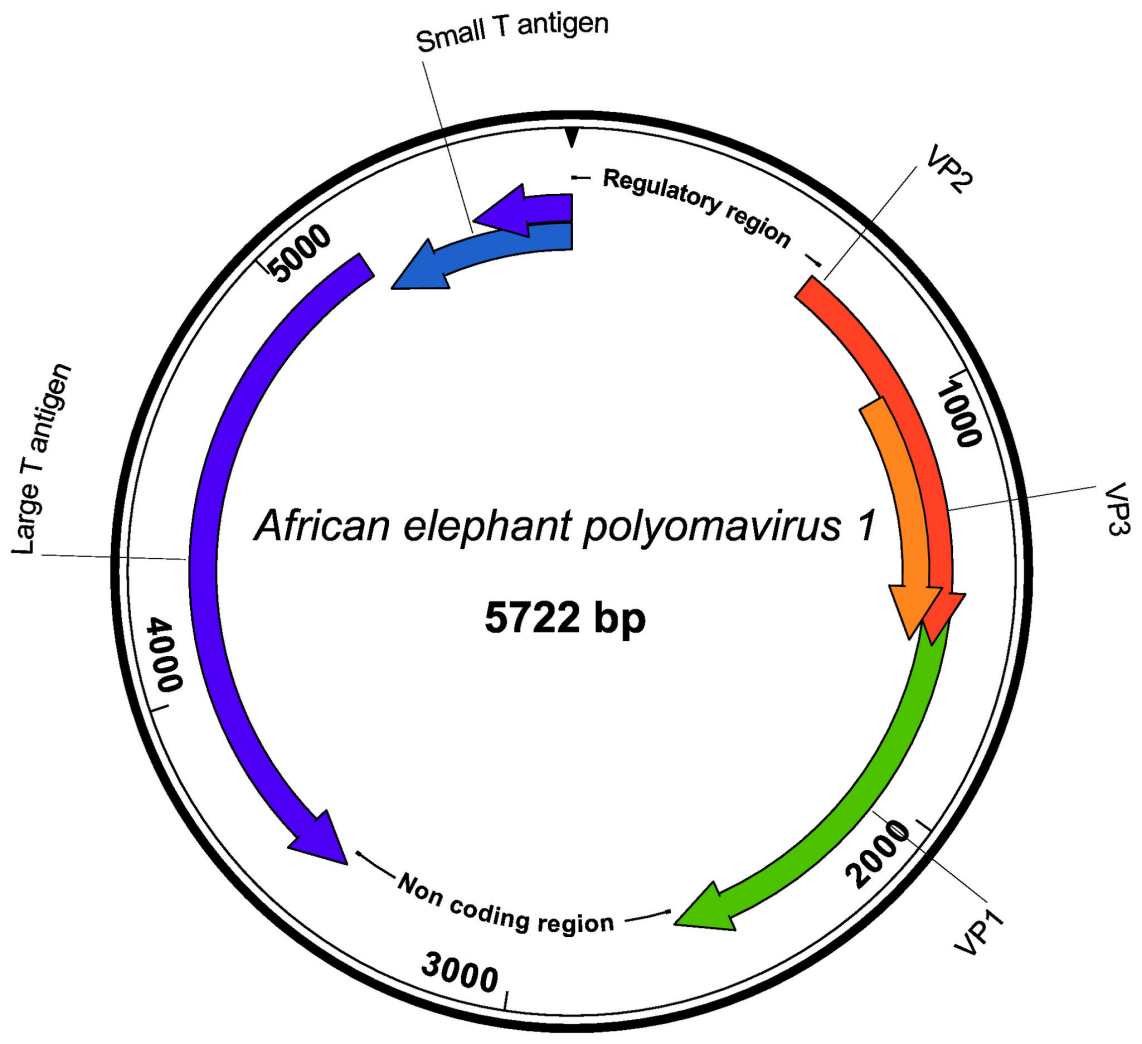
Schematic of the African elephant polyomavirus 1 (AelPyV-1) genome organization.

The purified RCA product was subjected to 454 sequencing and assembly of the produced reads resulted in 1 large contig of 5729 bp. Analysis of the contig and possible open-reading frames revealed that one or more errors were present in the contig. Subsequently the purified RCA product was subjected to Sanger sequencing to locate and correct the errors. Sanger sequencing was performed bi-directionally and the new contig produced had a total length of 5722 bp. This confirmed that 7 bases were added wrongly in the contig produced by 454 sequencing. All of the errors occurred in A or T homopolymer regions (5 bp or longer), a problem commonly reported for 454 sequencing [32].

The overall GC content of AelPyV-1 is 42.92%, which is similar to those of BK (39%), JC (40%), WU (39%) and other polyomaviruses [13]. The genome organization includes an early region coding on one strand for the small T antigen (STAg) and the large T antigen (LTAg), and a late region coding on the opposite strand for the capsid proteins VP1, VP2, and VP3, with a noncoding regulatory region between the beginning of the early region and the beginning of the late region, homologous to earlier described polyomaviruses [1] (Figure 2). With a length of 5722 bp, AelPyV-1 is the largest polyomavirus characterized at this moment, approximately 350 bp longer than the Merkel cell polyomavirus (5387 bp) [33]. The presence of a 854 bp long second noncoding region, between the end of the late region and the end of the early region, can possibly explain the large size of the AelPyV-1. In the proximal late region before the VP2 start codon, three potential small ORFs are present, which code for proteins with a length of respectively 43, 36 and 34 amino acids. Similarity analysis showed no similarity with already characterized agnoproteins. Due to the rather high content of basic amino acids (9 out of 43), however, the largest putative protein could possibly represent an open-reading frame for the agnoprotein. Conversely, even the largest ORF with 43 amino acids, is smaller than the already known agnoproteins, except for the putative Myotis polyomavirus agnoprotein, which is only 30 amino acids long [18]. Nonetheless, the basic nature and the genomic position are consistent with that of a mammalian polyomavirus agnoprotein [34]. Further research will be necessary to confirm whether one of the 3 putative agnoproteins is expressed and whether it is fully functional. The sizes of the proteins deduced from the genome sequences of AelPyV-1, their calculated molecular weights and isoelectric points are shown in Table 1. Analysis of the regulatory region showed an AT-rich region on the side of the late region of the putative replication origin, containing a possible AATAAA polyadenylation consensus sequence. Six copies of the consensus pentanucleotide LTAg binding site GAGGC, or the reverse complement GCCTG were present [35]. Three of them were found in a region with dyad symmetry, **GAGGC**TTAA**GCCTC**AG**GCCTC** , which might represent the possible core of the origin of replication. No regulatory elements could be found in the second noncoding region. BLAST similarity searches showed no similarity of this region with already characterized DNA sequences. Such an additional noncoding region is never reported before in polyomaviruses, but can be found in the family *Papillomavirus*, more specifically in species of the genus *lambdapapillomavirus*. It is possible that this second noncoding region represents an integration event with a piece of DNA of unknown origin.

**Table 1:** Size and position of predicted open reading frames (ORF) of AelPyV-1, the predicted molecular masses (kiloDalton), and the isoelectric point (pI) of the translated proteins.

ORF	Position	Length		MW (kDa)	pI
		nt	aa		
VP2	623 - 1615	992	330	36.4	6.61
VP3	956 - 1615	659	219	25.1	10.17
VP1	1485 - 2603	1118	372	40.6	6.04
LTAg	3458 - 5185	1975	658	75.8	7.00
	5474 - 5722				
STAg	5207 - 5722	515	171	19.2	9.20
URR^*^	1 - 622	n/a	n/a	n/a	n/a
NCR^**^	2604 - 3457	n/a	n/a	n/a	n/a

* Upstream regulatory region

** *Noncoding region*

A more detailed analysis of the amino acid sequences of AelPyV-1 revealed conserved sequences in functionally important regions of the encoded proteins. The proteins encoded by the 5 AelPyV-1 open-reading frames contain, although sometimes modified, the typical elements that are necessary to accomplish their function in the viral life cycle of polyomaviruses. The unspliced early mRNA of AelPyV-1 encodes the STAg, which is relatively short (171 aa) in comparison with other polyomaviruses. Furthermore, the CXCX_2_C consensus sequence for protein phosphatase 2A binding, which can be found in all mammalian polyomaviruses, is present in the STAg protein sequence. Like in most polyomaviruses, a single unspliced open-reading frame encodes the AelPyV-1 STAg. Only in the hamster polyomavirus and murine polyomavirus, the STAg transcript is spliced [13]. In polyomaviruses in general, the initial ~80 amino acids of the N-terminus of the STAg and LTAg are identical. The LTAg is generated by alternative splicing of the early mRNA transcript. In the AelPyV-1 early region, a conserved (CXXAG/GTXXX, with ‘/’ representing the breakpoint) splice donor site between base positions 245 to 254 ( **C**TG**AG**/**GT**TAG) was identified immediately after amino acid 83. Splicing to a conserved (TXTTXXAG/XTXCCXACXT) splice acceptor site between base positions 530 and 547 ( **T**C**TT**TT**AG**/G**T**G**CC**A**AC**T**T** ) would generate a predicted protein of 658 aa. Conserved features like the J-domain, which is important for efficient DNA replication and transformation, is found in the LTAg sequence. This domain contains the highly conserved HPDKGG box and a modified pRB-binding motif (IXCXD instead of LXCXE), crucial for DNA replication [9,36-38]. Furthermore, a zinc-binding motif (CX_2_CX_7_HX_3_HX_2_H) and an ATP-binding motif (GX_4_GK) are also present [39,40]. Finally, a putative nuclear localization sequence can be found in the LTAg protein sequence.

**Figure 3:**
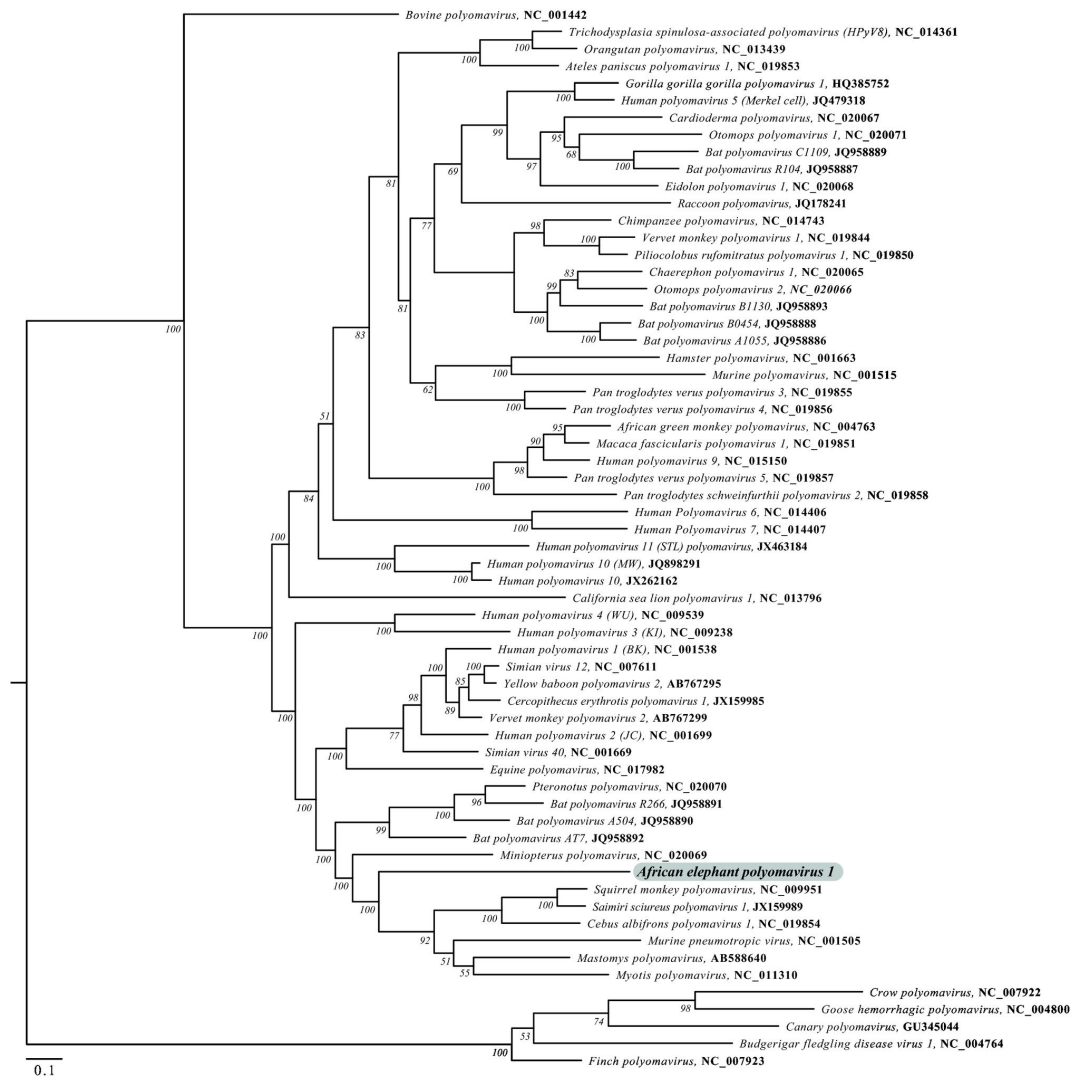
Nucleotide-based maximum likelihood phylogenetic analysis of the large T antigen open-reading frame of AelPyV-1 and 61 other polyomaviruses. The numbers at the internal nodes represent significant bootstrap support values, determined from 10,000 iterations. The scale bar indicates the genetic distance in nucleotide substitutions per site.

In the late region the major capsid protein VP1 is the largest and most conserved ORF, encoding a 372 aa long protein. In the N-terminal part of the VP1 protein sequence a nuclear localization sequence can be found, but the classic SV40 consensus sequence (KRKX_8_KKPK) is not conserved, which instead shows a KRPXRKPX_3_PR consensus sequence [41]. In the 330 aa long minor capsid protein VP2 sequence, the N-terminal consensus sequence MGX_4_S, which has been shown to mediate myristoylation in the Budgerigar fledgling disease polyomavirus capsid protein VP2 [42,43], is slightly modified to MGX_6_S. In polyomaviruses, the minor capsid protein VP3 is usually encoded by the same open-reading frame encoding VP2, by using an internal initiation codon. In AelPyV-1 the first methionine is positioned at amino acid 112 of the VP2 protein sequence and is considered to be the N-terminal amino acid of VP3, which results in a 219 aa long protein sequence.

**Figure 4:**
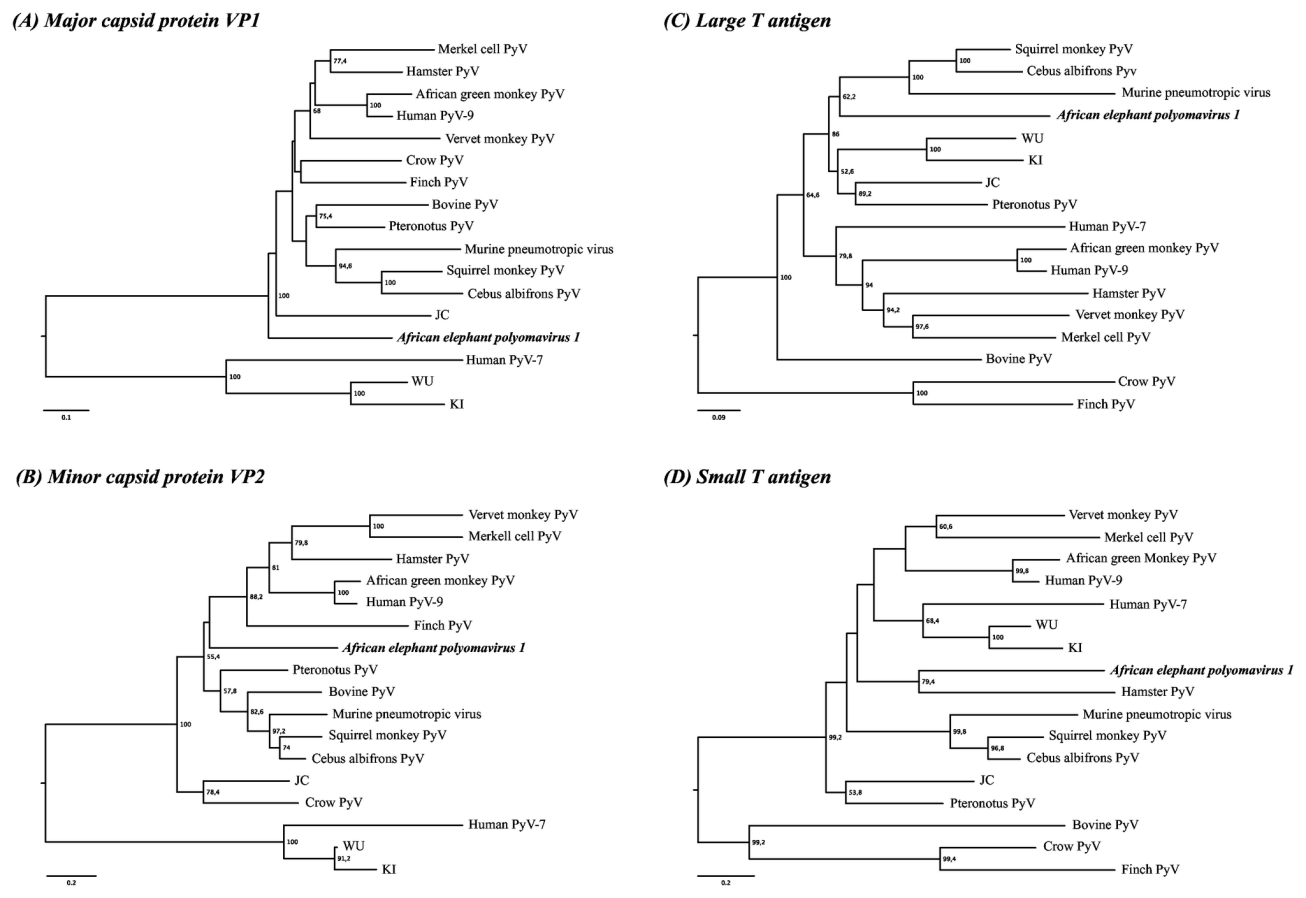
Amino acid-based neighbor-joining analysis of (A) the major capsid protein VP1, (B) the minor capsid protein VP2, (C) the large T antigen, and (D) the small T antigen. The numbers at the internal nodes represent significant bootstrap support values determined from 10,000 iterations. The scale bar indicates the genetic distance in amino acid substitutions per site. Genbank accession numbers: *African green* *monkey* *polyomavirus*, NC_004763; *Bovine* *polyomavirus*, NC_001442; *Cebus albifrons* *polyomavirus 1*, NC_019854; *Crow* *polyomavirus*, NC_007922; *Finch* *polyomavirus*, NC_007923; *Hamster* *polyomavirus*, NC_001663; *Human* *polyomavirus 2* (JC), NC_001699; *Human* *polyomavirus 3* (KI), NC_009238; *Human* *polyomavirus 4* (WU), NC_009539; *Human* *polyomavirus 5* (Merkel cell), JQ479318; *Human* *polyomavirus 7*, NC_014407; *Human* *polyomavirus 9*, NC_015150; *Murine* *pneumotropic* *virus*, NC_001505; *Pteronotus polyomavirus*, NC_020070; *Squirrel* *monkey* *polyomavirus*, NC_009951; *Vervet monkey* *polyomavirus 1*, NC_019844. PyV: Polyomavirus.

Figure 3 depicts the phylogenetic analysis of the polyomavirus LTAg based on a maximum likelihood analysis of the nucleotide sequences. AelPyV-1 clusters together with a heterogeneous group of bat (5), rodent (4) and monkey (2) polyomaviruses. Neighbor joining analysis of the VP1, VP2, STAg and LTAg proteins demonstrated that AelPyV-1 is highly divergent from other polyomaviruses. Analysis of VP1 showed that AelPyV-1 clustered separately from the cluster of Orthopolyomaviruses (Figure 4A). Based on VP2 and STAg protein sequences, AelPyV-1 clustered with the heterogeneous group of vervet monkey, merkel cell, hamster, African green monkey, human PyV9 and Finch polyomaviruses (Figure 4B), and the hamster polyomavirus (Figure 4D) respectively. The LTag protein analysis revealed similar clustering as seen with the maximum likelihood based on LTag nucleotide sequences (Figure 4C).

Using a method that specifically amplifies the circular genome of polyomavirus genomes, we were able to recover the entire genome of a new polyomavirus out of a protruding hyperplastic fibrous lesion of an African elephant. We suggest to provisionally naming the virus African elephant polyomavirus 1. An important issue to this regard is whether AelPyV-1 is the causal infectious agent of the aforementioned fibrous lesions. Mammalian polyomaviruses infections are highly common during childhood and adolescence [44]. Nevertheless these presumably lifelong persistent viruses are not generally associated with acute disease in their natural non-immunocompromised hosts [18]. On the other hand, mammalian polyomaviruses are known to induce tumors after inoculation into non-permissive laboratory rodents [45] and recently, the human polyomavirus, Merkel cell polyomavirus, has been linked to the development of skin carcinoma [46]. Moreover, polyomaviruses of birds have been known to cause acute and chronic diseases in several species of birds [47,48].

Intriguingly, Jacobson and colleagues described proliferative cutaneous lesions in a herd of captive African elephants in 1986 [49]. Like the presented case, these lesions were predominantly located on the trunks of the animals and often progressed to large fibromatous lesions. Although the authors point to papillomaviruses as the causative agent, no viral DNA or viral papillomavirus antigens were detected. Moreover, no papillomavirus virions were found with electron microscopy in the elephant lesions. The case report significantly resembles our findings, which may point to a common infection origin. It is clear that further studies are needed to elucidate if the African elephant is the natural host of AelPyV-1 and to assess the potential association with the described disease symptoms.
